# Lifestyle advice follow-up improve glycemic control, redox and inflammatory status in patients with type 2 diabetes

**DOI:** 10.1186/s40200-014-0122-1

**Published:** 2014-12-17

**Authors:** Nadia Mahdad, Farida Ouda Boukortt, Zakaria Benzian, Malika Bouchenak

**Affiliations:** Laboratoire de Nutrition Clinique et Métabolique, Faculté des Sciences de la Nature et de la Vie, Université d’Oran, BP 1524 El M’Naouer, 31 000 Oran, Algeria; Clinique Laribère, Centre Hospitalo-Universitaire CHU, 31 000 Oran, Algeria

**Keywords:** Type 2 diabetes, HbA1c, Redox status, Resistin, TNF-*α*

## Abstract

**Background:**

The dietary composition associated to physical activity could play a significant role in improving insulin sensitivity and reducing risk of diabetes and its complications. This study was designed to investigate whether glycemic control, redox and inflammatory status impairments in patients with type 2 diabetes (T2D), were improved after 90 (d90) and 180 (d180) days follow-up of nutritional advices.

**Methods:**

Patients with T2D (n = 85) aged of 50 ± 8 years (Female/Male, 45/40), treated with oral antidiabetics (OAD) alone, with a body mass index (BMI) of 26 ± 2, were recruited. At the beginning of the study (d0), patients were instructed to follow-up nutritional advices adapted to T2D, and 30 to 45 min of walking per day. Assays were realized at d90 and d180 of follow-up. Data were compared by student ‘t’ test and Pearson’s correlation coefficients were determined between biochemical parameters and nutritional advices follow-up.

**Results:**

Reduced glycated haemoglobin (HbA1c), glucose and total cholesterol (TC) were noted in patients with T2D, at d90 and d180 compared to d0. Thiobarbituric acid reactive substances (TBARS) and hydroperoxyde levels were lower at d90 and d180 than d0. Serum nitric oxide (NO) was decreased at d180 compared to d0 and d90. In erythrocytes, superoxide dismutase (SOD) activity increased by 7% at d180 *vs* d0. Moreover, activity of glutathione peroxidase (GPx) enhanced (*P < 0.05*), whereas that of glutathione reductase (GRed) decreased (*P < 0.001*) at d90 *vs* d0. Resistin values were lower at d180 than d0 and d90 (*P < 0.001*). A progressive decrease in tumor necrosis factor-α (TNF-α) was observed at d90 and d180 *vs* d0.

**Conclusion:**

Nutritional advices associated to physical activity improve glycemic control, serum TC, redox and inflammatory status in T2D, in particular after 3 months of counseling. However, these results need to be supported with a longer dietary treatment and more rigorous control during the follow-up.

## Background

Dietary and lifestyle approaches have a high potential for the primary prevention of type 2 diabetes (T2D) [1]. This disease is a multistage process that begins as insulin resistance (IR), characterized by body inability to use properly its own insulin, and ends with exhaustion of the insulin-producing pancreatic ß-cells, thereby leading to hyperglycaemia [2]. Several factors are implicated in T2D development, including obesity, family history, physical inactivity and inherited factors [2]. Moreover, dietary composition could play a significant role in improving insulin sensitivity and reducing risk of diabetes and its complications [3]. Indeed, cardiovascular diseases (CVD) are the major cause of morbidity and mortality in T2D [4], knowing that cardiovascular risk is threefold higher in T2D than in general population [5]. In Algeria, the prevalence of T2D was about 10% in 2010 [6].

In T2D, the oxidative stress (OS) acts as a mediator of IR, and its progression to glucose intolerance and installation of the pathology, possibly contributes to the rise of several micro- and macro-vascular complications associated with diabetes [7]. In conditions of severe OS, cell damage occurs with decreased pancreatic ß-cell function, which is due to low expression of antioxidant enzymes [8].

However, clinical studies have also shown that specific antioxidant concentrations in plasma and erythrocytes of diabetes patients are reduced [9]. Indeed, superoxide dismutase (SOD), glutathione peroxidase (GPx) and catalase (CAT) activities scavenging reactive oxygen species (ROS), were decreased in patients with T2D. Thus, the improvement in oxidative stress status can contribute to diabetes management [10].

Inflammation is considered to be a key regulator of T2D pathogenesis, but the mechanism, by which it acts, is still unknown. Tumor necrosis factor (TNF-α) is an inflammatory cytokine implicated in metabolic disorders, including obesity and IR. Several studies have shown high levels of interleukin-6 (IL-6) and TNF-α among individuals with clinically diagnosed diabetes [11]. Moreover, authors reported increased resistin levels in association with obesity and IR in T2D [12], whereas other study observed no change in resistin levels under such conditions [13]. Data have shown that circulating resistin levels were involved in promoting adiposity, but had no effect on IR degree. These observations suggested that the role of resistin in the pathogenesis of diabetes remained controversial [14].

The purpose of the present study was to investigate whether TD2 impaired glycemic control, redox and inflammatory status, and their improvement after 3 and 6 months follow-up of nutritional advices.

## Methods

This study was conducted from November 2009 to June 2011 in the Hospital University Establishment (HUE) (Internal Medicine) and polyclinics of Oran (west Algeria). Patients with T2D since 9 ± 3 years (n = 85) (sex ratio F/M, 45/40), with a mean age of 50 ± 8 years treated during 8 ± 1 years with oral antidiabetics (OAD as biguanides and/or sulfamides) only, with a body mass index (BMI) (kg/m^2^) of 26 ± 2, fasting glycemia ≥ 7 mmol/L, triglyceridemia < 2 mmol/L, cholesterolemia < 5 mmol/L and low-density lipoproteins-cholesterol (LDL-C) < 3.5 mmol/L.

Patients with other diabetes forms or T2D complicated or treated with insulin, obesity, renal failure, hepatic and thyroïd diseases, smoking, consuming of alcohols were excluded. The clinical characteristics of patients are presented in Table 1.

**Table 1:** **Characteristics of patients with type 2 diabetes**

	**T2D**
Sex ratio (F/M)	45/40
Age (year)	50 ± 8
Weight (kg)	70 ± 9
BMI (kg/m^2^)	26 ± 2
Waist circumference (cm)	89 ± 8
Diabetes duration (year)	9 ± 3
Glycemia (g/L)	1.68 ± 0.65
HbA1c (%)	9.8 ± 1.9
Total cholesterol (mmol/L)	4.16 ± 0.70
Triglycerides (mmol/L)	1.15 ± 0.57
LDL-C (mmol/L)	1.20 ± 0.33

Data are mean ± SD of 85 patients. BMI: body mass index (weight (kg)/height (m^2^)). HbA1c: glycated haemoglobin. LDL-C: low-density lipoproteins-cholesterol.

This study was approved by the Institutional Ethic Committee for Research on Human Subjects of the University of Oran. The protocol and the aim of the study were explained to the patients and the investigation was conducted with their written informed consent.

Nutritional counseling was designed to cover the nutritional needs and maintain life quality of patients. The recommended daily energy intake was 2000 Kilocalories (Kcal) with 250 g of carbohydrates distributed over different meals in the day i.e. breakfast, morning snack, lunch, afternoon snack and dinner. The advice given promoted the consumption of fish meal, fruits and vegetables (FFV). Consumption of a dairy product at all the three main meals daily, vegetables (2 times/d), fruits (2–3 times/d), olive oil (2 table spoons/d), whole bread daily at each meal, fish (2–3 times/week), with limited eggs consumption (4–5 eggs/week) and moderate amounts of red or white meats, sources of animal proteins, at lunch and dinner were recommended. Moreover, patients were instructed for 30 to 45 min of walking per day. The follow-up was performed at d90 and d180.

Height and weight were performed using stadiometer and balance (Weighting Scale ZT 220, China). Waist circumference (WC) was measured mid-way between the lateral lower rib margin and the iliac crest (ombilicus).

Blood was drawn after a 12-hour overnight fasting by antecubital venipuncture, at d0, d90 and d180, after lifestyle advices. Serum and erythrocytes were collected by low-speed centrifugation at 1000 g for 15 min. Serum was preserved with 0.1% ethylene–diamine-tetraacetic acid-disodium (Na_2_-EDTA). Erythrocytes were washed two times with 0.9% sodium chloride (NaCl) solution, and four times with cold water. Erythrocytes lysates were preserved with 0.5% sodium dodecylsulfate (SDS, Sigma, USA). Serum and erythrocytes lysates were stored at -70°C until assays.

Serum glucose was measured by enzymatic colorimetric test (kit Biocon, Germany). Serum insulin was determined by an *in vitro* enzyme-linked immunosorbent assay (ELISA) (kit Abcam, Cambridge, USA) for the quantitative measurement of human serum insulin and proinsulin. HbA1c was estimated by chromatographic spectophotometric test (kit Biocon, Germany). Homeostatic model assessment insulin resistance (HOMA-IR) index (IR = fasting serum insulin (μU/mL) × fasting plasma glucose (mmol/L)/22.5) was determined [15].

Serum total cholesterol (TC), triglycerides (TG) and uric acid (UA) levels were measured by enzymatic colorimetric tests (kits Biocon, Germany). Albumin was determined by colorimetric method (Kit Biolabo, France).

Serum and erythrocytes lipid peroxidation was estimated by measuring thiobarbituric acid reactive substances (TBARS) [16]. The colored complex formed between malondialdehyde (MDA) and thiobarbituric acid (TBA) had maximum absorbance at 532 nm. The serum hydroperoxyde concentrations were determined by measuring cumin hydroperoxyde (EqCuOOH) by comparison of the straight line [17]. The absorbance was measured at 560 nm. Oxidized proteins were estimated by measuring carbonyl concentrations, using the 2,4-dinitrophenylhydrazine [18]. The absorbance was measured at 270 nm. Nitric oxide (NO) determination was performed using Griess reagent (sulfamide and n-naphtyl-ethylene diamine) [19]. Serum was clarified by zinc sulfate solution and nitrate (NO_3_) was then reduced to nitrite (NO_2_) by cadmium, overnight, at 20°C, under shaking. Serum was added to the Griess reagent and incubated for 20 min at 24°C. The absorbance was measured at 540 nm.

SOD, GPx (EC 1.11.1.9) and GRed (EC 1.6.4.2) activities in erythrocytes were determined by kits (Sigma, USA). SOD activity was assessed at 450 nm by measuring the dismutation of superoxide radicals generated by xantine oxidase and hypoxantine. The oxidation of glutathione (GSH) to oxidized glutathione (GSSG) was catalysed by GPx, which was then coupled to recycling of GSSG back to GSH utilizing GRed and NADPH (reduced β-nicotinamide adenine dinucleotide phosphate) (Sigma, USA). The activity was measured by the decrease in absorbance caused by the oxidation of NADPH at 340 nm using an extinction coefficient (ε^mM^) of 6.22 for NADPH. Catalase (CAT) (EC. 1.11.1.6) catalyses the decomposition of hydrogen peroxide to water and oxygen at 240 nm [20].

TNF-α was assayed by enzyme immunometric assay (EIA) kit (Cayman Chemical’s ACE™ EIA kit, USA) permitting TNF-α measurements within the range of 0–250 pg/ml. The lower limit of detection was 3.9 pg/mL for TNF-α. The human resistin was determined by EIA (SPI Bio – Bertin Pharma Branch kit, France). The normal range was established for 8.1 ± 4.1 ng/ml.

### Statistical analysis

The non-parametric student ‘*t*’ test (STATISTICA 5.0.) was used to compare mean differences between d0, d90 and d180. Relationships between all the biochemical parameters and the nutritional advices follow-up were determined by the Pearson’s correlation coefficient. *P values <0.05* were considered significant.

## Results

Among 85 patients with T2D, at d0 and after 3 months of nutritional advices (d90), only 35 patients completed this study at d180. The remaining patients became hypertensive and/or were treated by insulin. There was no significant difference in studied parameters when related to a gender.

There was no significant difference in body weight, BMI and WC of T2D patients at d90 and d180 *vs* d0 (Table 2). HbA1c value decreased by 40% at d90 and by 20% at d180 compared to d0. A significant reduction in glucose concentration at d90 *vs* d0 (19%) and d180 *vs* d0 (12%) was noted, whereas this value increased by 7% at d180 *vs* d90. There was no significant difference in insulinemia and HOMA-IR, after lifestyle advices (Table 2).

**Table 2:** **Clinical and biochemical parameters in TD2 patients after nutritional advices**

	**d0**	**d90**	**d180**
	**n = 85**	**n = 85**	**n = 35**
Weight (kg)	70 ± 7	70 ± 9	69 ± 9
BMI (kg/m^2^)	26 ± 2	25 ± 2	24 ± 2
Waist circumference (cm)	89 ± 8	86 ± 7	88 ± 5
Hb1Ac (%)	9.8 ± 1.9	6.4 ± 1.7^***^	8.4 ± 1.0^§^
Glucose (mmol/L)	7.71 ± 1.50	6.27 ± 1.56^**^	6.76 ± 0.77^ǂ§^
HOMA-IR	13.10 ± 4.32	12.53 ± 4.26	8.75 ± 1.20
Total cholesterol (mmol/L)	4.16 ± 0.70	3.47 ± 1.10^**^	3.09 ± 1.30^§§^
Triglycerides (mmol/L)	1.15 ± 0.57	0.73 ± 0.35^**^	1.26 ± 0.53^ǂǂ^

Values are means ± SD. d0, beginning of study; d90: 90 days and d180: 180 days after initiating lifestyle advices. Data were analyzed using ‘t’ test. ^***^ *P* < 0.001, ^**^ *P* < 0.01 d90 *vs* d0; ^§§^ *P* < 0.01, ^§^ *P* < 0.05 d180 *vs* d0; ^ǂ^ *P* < 0.05, ^ǂǂ^ *P* < 0.01 d180 *vs* d90.

Compared to d0, serum TC concentrations were lowered by 17% and 26% at d90 and d180, respectively. Moreover, TG concentrations in patients with T2D were reduced by 36% at d90 compared to d0, whereas, these values increased by 42% at d180 compared to d90 (Table 2).

In serum, TBARS values were 1.34-fold lower at d90 and d180 than d0. Moreover, compared to d0, hydroperoxyde concentrations were l.6- and 2-fold lower at d90 and d180, respectively. There was no significant difference in carbonyl values after initiating lifestyle advices. Serum NO was 1.4- and 1.2-fold lower at d180 than d0 and d90, respectively. UA values were 1.34-fold higher at d180 than d0 (Table 3). Albumin concentration was significantly enhanced at d90 *vs* d0 (P < 0.001).

**Table 3:** **Redox status and inflammation markers in T2D patients after lifestyle advices**

	**d0**	**d90**	**d180**
	**n = 85**	**n = 85**	**n = 35**
**Serum**			
TBARS (μmol/ml)	6.53 ± 1.90	4.87 ± 1.31^*^	4.90 ± 1.77^§^
Hydroperoxydes (mmol/ml)	0.31 ± 0.06	0.19 ± 0.06^***^	0.16 ± 0.06^§§§^
Carbonyls (nmol/mg prot)	1.07 ± 0.73	0.85 ± 0.41	0.80 ± 0,35
NO (mmol/ml)	2.26 ± 0.41	1.91 ± 0.36	1.57 ± 0.14^§**ǂ**^
Uric acid (μmol/L)	229.52 ± 67.10	238.10 ± 77.12	308.33 ± 118.52^§^
Albumin (g/L)	36.60 ± 12.50	51.00 ± 7.00^***^	46.35 ± 5.43
**Erythrocytes**			
SOD (U/gHb)	2430.11 ± 824.38	2045.37 ± 710.10	2622.00 ± 541.14^ǂ^
GPx (U/gHb)	3.14 ± 1.98	5.17 ± 2.63^*^	5.45 ± 3.08
GRed (U/gHb)	1.97 ± 0.61	1.15 ± 0.45^**^	1.52 ± 0,60
CAT (U/g Hb)	18124.00 ± 5549.01	15128.00 ± 5067.32	16099.52 ± 5354.30
Resistin (ng/ml)	15.78 ± 2.24	13.95 ± 0.82	5.10 ± 1.20^§ǂǂ^
TNF-α (pg/ml)	3.44 ± 1.19	2.33 ± 0.81^*^	1.89 ± 0.9^§^

Values are means ± SD. d0, beginning of study; d90 and d180, 90 and 180 days after initiating lifestyle advices. SOD, superoxide dismutase; GPx, glutathione peroxidase; GRed, glutathione reductase; CAT, catalase. TNF-α, tumor necrosis factor α. Data were analyzed using ‘t’ test. ^*^ *P* < 0.05, ^**^ *P* < 0.01, ^***^ *P* < 0.001: d90 *vs* d0. ^§§§^ *P* < 0.001, ^§^ *P* < 0.05 d180 *vs* d0. ^ǂ^ *P* < 0.05, ^ǂǂ^ *P* < 0.01: d180 *vs* d90.

In erythrocytes, SOD activity increased by 7% in T2D at d180 *vs* d0. There was no significant change in CAT activity of T2D patients during the follow-up, whereas, increased GPx activity by 39% was observed at d90 compared with d0. However, GRed activity was reduced by 42% at d90 *vs* d0.

Resistin values were 3- and 2.7-fold lower, in T2D at d180 than d0 and d90 respectively. Moreover, TNF-α concentrations were 1.5- and 1.8-fold decreased at d90 and d180, after nutritional advices than d0.

Relationships were found between HbA1c values and BMI, TBARS, CAT and GRed. HOMA-IR was correlated negatively with UA and positively with NO and resistin. However, inverse relationships were noted between resistin and UA and SOD, and positive correlations were observed with TG, NO and TNF-α (Table 4).

**Table 4:** **Correlation studies between HbA1c, HOMA-IR and resistin and various parameters in T2D patients**

	**HbA1c**	**HOMA-IR**	**Resistin**
T2D duration	0.050	−0.130	−0.010
IMC	0.410^**^	0.230	0.050
WC	0.050	0.850	0.078
Glucose	0.420^***^	0.010	−0.100
TG	0.190	−0.180	−0.190
TC	0.100	0.130	0.410^***^
TBARS	0.300^*^	−0.007	0.370
Hydroperoxydes	0.130	0.270^*^	0.004
NO	0.055	0.270^*^	0.400^**^
carbonyls	0.130	−0.030	0.083
Uric acid	0.100	−0.290^*^	−0.250^*^
Albumin	−0.210	−0.051	−0.060
SOD	0.085	−0.600	−0.320^*^
CAT	0.263^*^	0.070	0.017
GPx	−0.160	−0.050	−0.010
GRed	0.330^**^	0.100	−0.040
TNF-α	0.180	0.012	0.400^***^
Resistin	0.005	0.350^**^	

The values expressed as Pearson’s correlation coefficients.

*Correlation is significant at *P < 0.05.* **Correlation is significant at *P < 0.01.* ***Correlation is significant *at P < 0.001*.

## Discussion

The aim of the present study was to determine if the nutritional advices improved glycemic control, redox and inflammatory status in T2D patients treated with OAD only.

After nutritional advices based on eating FFV and carbohydrates distribution at each meal, T2D patients showed no significant difference in BMI and WC, whereas, large studies showed that excessive abdominal fat deposition is an important risk factor for T2D [21] and that high WC is a better predictor than BMI [22].

Lowering HbA1c in T2D decreased the CVD risk and all mortality causes [23]. After d90 and d180 of nutritional advices, HbA1c and glucose values lowered significantly in T2D, showing relationships between glycemic control and BMI. This improvement was probably due to the recommended FFV consumption associated with physical activity practice. Indeed, a moderate effect of regular physical exercise with a mean HbA1c decrease, without significant weight loss, was observed despite favorable changes in body weight composition [24]. Similar results showed, that three months consumption of fruits, reduced blood glucose and HbA1c levels, whereas, no significant changes were noted in BMI and WC [25]. At d180 *vs* d90, increased serum glucose level was noted, without change in serum insulin concentrations, whereas HOMA-IR had a tendency to decrease but not significantly. The most plausible explanation, for this phenomenon, was that d180 corresponded to summer period with many festive meals, and during holidays, people were customarily physically inactive and were prone to forget diet recommendations. Indeed, it has been shown that seasons can influence fasting glucose and HbA1c in T2D patients [26]. Our T2D patients were instructed to consume 2 table spoons of olive oil daily and fish intake 2-times weekly. Indeed, data have shown that n-3 poly-unsaturated fatty acids (PUFAs) from fish may decrease IR through several mechanisms, such as a decrease in plasma TG, and perhaps small dense lipoproteins [27], and the consumption of mono-unsaturated FA (MUFA)-rich diet improve IR in T2D [28].

A high intake of dry or fresh vegetables and fruits was associated with a better control regulation of lipid profile [29]. In the present study, nutritional advices reduced serum TC and TG at d90 *vs* d0. Indeed, dietary MUFA (oleic acid) decreased LDL-C and TG concentrations, without lowering high-density lipoproteins-cholesterol (HDL-C) [30]. Moreover, the improved lipid profile was probably due to the daily regular 30 to 45 min walking by our patients. Indeed, physical activity decreased TG and LDL particles and enhanced HDL-C [31].

T2D is associated with increased oxidative stress, contributing to this pathogenesis [32], by increasing serum TBARS [33] and hydroperoxides [34]. At d180 and d90 compared to d0, TBARS and hydroperoxides concentrations were significantly reduced in our T2D patients. Furthermore, the lowered lipid peroxidation could be due to the improved glycemic control, and therefore to a lesser oxidative stress, showing relationships between TBARS and HbA1c. Likewise, consumption of fruits and vegetables, rich in antioxidants, could play an important role in protecting our patients from oxidative stress generated by T2D.

A high level of plasma protein carbonyls has been reported in T2D patients [35], and can also originate from a loss of albumin antioxidant capacity, knowing that plasma albumin, *via* its thiol groups, is the main extracellular antioxidant molecule [36]. In our study, serum carbonyl values had a tendency to decrease, whereas serum albumin value was enhanced, after lifestyle advices, suggesting that improved glycemic control can reduce oxidative stress, and therefore carbonyl amounts, without altering albumin properties.

Some controversies about the NO role are noted in the IR pathogenesis [37]. Moreover, NO, a metabolite of L-arginine to L-citrulline conversion by endothelial NO synthase, has a favorable effect on inflammation [38], and oxidative stress [39]. A decrease in NO values at d180 *vs* d0 and d90 was noted and was positively correlated with IR and serum resistin.

Hyperuricaemia is associated with IR [40], which is mediated partly by inflammation and oxidative stress [41]. However, our results showed, at d180 *vs* d0, that high UA level was inversely related to HOMA-IR and resistin. Indeed, this result suggested that uric acid value was efficient to reduce IR and inflammation in T2D.

Besides the important lipid peroxidation in T2D patients, an increase in SOD activity was observed in such individuals [42]. In our T2D patients, erythrocytes SOD activity increased at d180 *vs* d0 (*P < 0.05*), suggesting a possible adaptative response, probably due to a high production of superoxide anion (O_2_^−^), which would lead to an increased hydrogen peroxide (H_2_O_2_) production [42]. This latter has been reported to inactivate SOD, and superoxide anion radical inactivates CAT [43], and GPx [44]. However, there was no significant difference in the both enzyme activities at d180 *vs* d0. In our T2D patients, GRed activity was lower, while GPx was higher, at d90 *vs* d0. In contrast, diabetic complications involved lowered GPx activity, which could also be due to decreased glucose-6-phosphate dehydrogenase activity, in erythrocytes [45], leading to reduced NADPH production, required for recycling processes of GSSG to GSH, which was the GPx substrate [45].

There is a genetic evidence to support relationships between human inflammation, in particular resistin and obesity or IR [46]. In the present study, lowered serum resistin values, at d180 *vs* d90 and d0, were observed, and were positively correlated with IR. Moreover, TNF-α was reduced significantly in T2D, after nutritional advices, compared to the beginning of the study. High levels of inflammatory cytokines appear in early stage of T2D, and predict the development of this disease, through lowering insulin sensitivity [47]. Indeed, a highly significant relationship between resistin and TNF-α, and a negative correlation between resistin and SOD were observed. Moreover, observational studies have shown an inverse association between dietary total antioxidant capacity and inflammation markers [48], whereas, n-3 PUFA supplementation may potentially affect these markers, in patients with T2D [49]. In our study, recommended FFV could contribute probably to decreased serum resistin and TNF- *α*, and thus protecting the organism from proinflammatory cytokines deleterious effects.

## Conclusion

The nutritional advices follow-up i.e. FFV consumption, and carbohydrates distribution at each meal, associated to a regular physical activity practice, improve glycemic control and serum total cholesterol in T2D. Moreover, a low lipid peroxidation and a high antioxidant defense are noted, involving a less oxidative stress induced by T2D, on the one hand, and reduced serum resistin and TNF-*α* are in favor of a low progression to complications, on the other hand. However, these results need to be supported with a regular dietary treatment and more rigorous control during the follow-up.
